# Dabigatran and Edoxaban in the Repair of an Abnormal Cleft in the Right Wall of the Ascending Aorta: A Case Report

**DOI:** 10.7759/cureus.101802

**Published:** 2026-01-18

**Authors:** Hidekazu Takeuchi

**Affiliations:** 1 Internal Medicine (Cardiology), Takeuchi Naika Clinic, Ogachi-Gun, JPN

**Keywords:** aortic aneurysm, aortic dissection, ascending aorta, atherosclerosis, dabigatran, edoxaban, medial degeneration

## Abstract

The appearance of a cleft may be the clear start of an aortic aneurysm and aortic dissection (AA/AD). If such small clefts can be identified and cured at an early stage, a new treatment could be developed for AA/AD prevention. In this case report, linear white (high echo intensity) thrombi extending from the right upper pulmonary vein (RUPV) penetrated the wall of the ascending aorta (AAo), resulting in a cleft in the AAo wall in an area with possible medial degeneration. Additionally, we show that dabigatran and edoxaban partially resolved the linear thrombi and cured the cleft.

## Introduction

Ancient Egyptian and Mayan people had atherosclerosis and medial degeneration of the aortic wall, calling into question the traditional concept of atherosclerosis [1]. Medial degeneration, which is characterized by smooth muscle cell disorganization, smooth muscle cell nuclear loss, elastic medial fibrosis, and fiber thinning, was reported to occur in most of the examined people, although the relationships between medial degeneration and atherosclerosis were not statistically significant [2].

Cells affected by medial degeneration are known to contain tissue factor (TF), which initiates thrombus formation [3]. The mechanisms underlying the development of medial degenerative changes in humans are unclear. In 2025, I described the presence of a mass on the ascending aorta (AAo) wall, which appeared to be connected to right upper pulmonary vein (RUPV) thrombi through linear white (high reflectivity) thrombi [4,5]. The mass on the AAo wall could have been a mass of medial degenerative cells, with thrombi in the AAo.

Aortic aneurysm and aortic dissection (AA/AD) are severe and fatal aortic disorders with unknown etiology. Sudden rupture usually results in unavoidable death; therefore, clarifying the pathogenesis of AA/AD is crucial. Mural thrombi are common in patients with AA/AD [6].

Studies of thrombi retrieved from patients with acute ischemic stroke (AIS) have shown that these thrombi have calcifications and endothelial cells [7], indicating that they are chronic or old and associated with some type of vessel. Patients with AIS or acute myocardial infarction (AMI) develop chronic thrombi before the occurrence of AIS or AMI; however, few reports have described candidates for chronic thrombi.

I have reported that most elderly patients with hypertension, dyslipidemia, and/or type 2 diabetes mellitus (T2DM) have pulmonary vein (PV) thrombi according to enhanced computed tomography (CT) and transesophageal echocardiography (TEE) [4,5,8], and that these thrombi sometimes have shadows on TEE, indicating the presence of calcifications [4,5]. These PV thrombi can release other thrombi, which can cause AMI or AIS. Additionally, I reported that RUPV thrombi and AAo thrombi seemed to be connected through linear white thrombi with a small mass on the AAo wall in the connecting areas. The wall in the area of the mass appeared to have lost some of its normal structure, indicating that the mass was affected by medial degeneration [4,5].

In this case, linear white thrombi appeared to penetrate the AAo wall, revealing the presence of a cleft, and the effects of dabigatran (thrombin inhibitor) and edoxaban (Factor Xa inhibitor) treatment were good.

## Case presentation

The patient was a 68-year-old female who had dyslipidemia and hypertension, as well as chronic left atrial appendage (LAA) thrombi and PV thrombi, as assessed by enhanced CT and TEE. Her breath sounds were normal, with no crackles or wheezes. Her heart sounds were clear, and the rhythm was regular, without audible murmurs or friction sounds. Her blood pressure was 132/86, her pulse was 60 and regular, and her respiration count was 13. Her brain natriuretic protein (BNP) concentration was 22.4 pg/mL (normal: <18.4 pg/mL).

Electrocardiography (ECG) demonstrated a normal sinus rhythm, a normal axis, and counterclockwise rotation. The serum D-dimer concentration was 0.7 μg/ml (normal: <1.0 μg/ml), the activity of protein S was 91% (normal: 74%-132%), and the activity of protein C was 114% (normal: 64%-135%).

**Figure 1:**
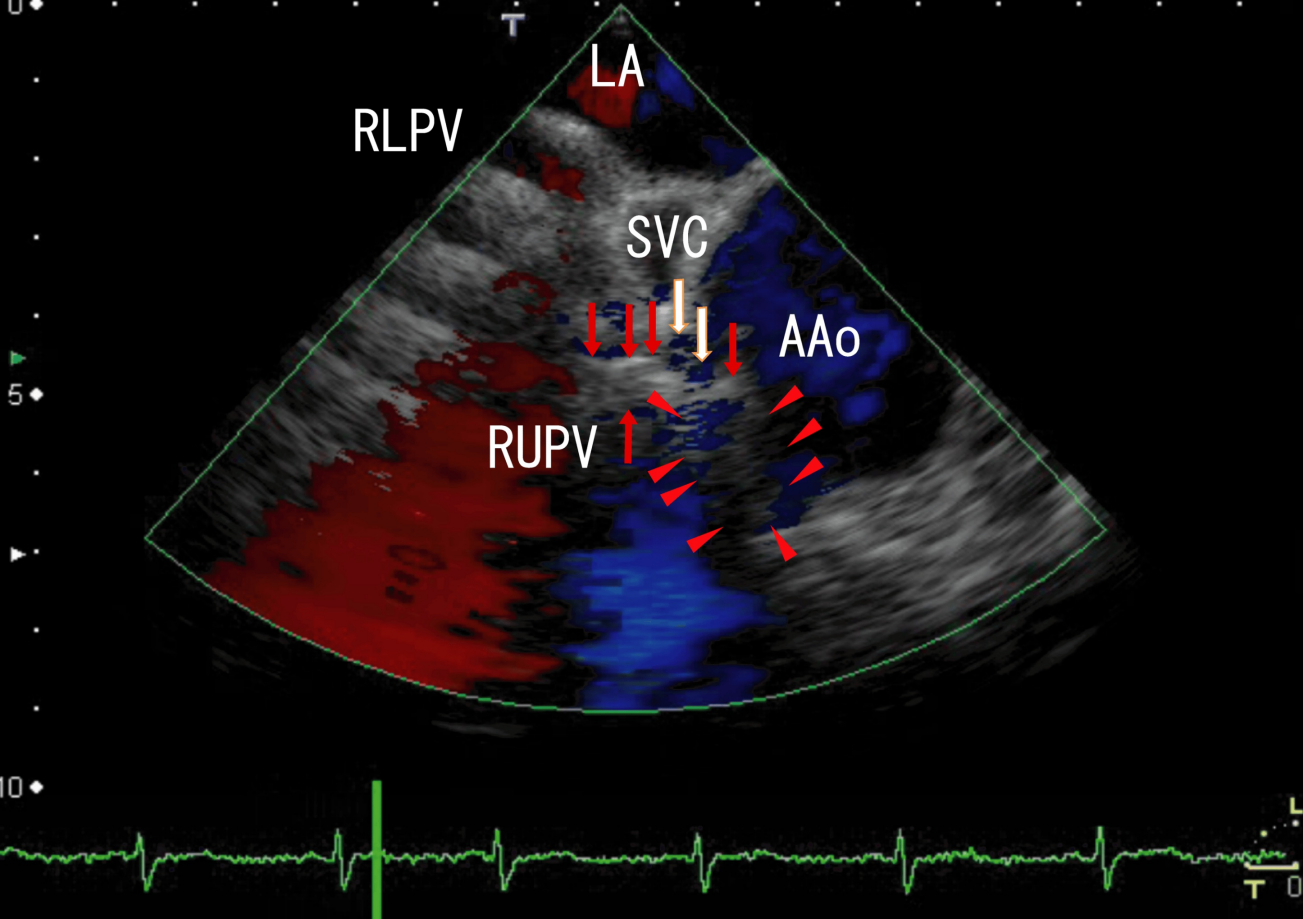
Transesophageal echocardiography (TEE) images showing ascending aorta (AAo) thrombi and linear white thrombi The TEE revealed thrombi in the AAo and linear white thrombi from the RUPV into the AAo (red arrows), and the linear white thrombi were situated in the anterior of the SVC. There were small blue areas between the end of the AAo wall and the linear white thrombi on the AAo wall (white arrows), indicating a cleft in the AAo wall and penetration of the AAo wall by the thrombi. Approximately 2 cm of the right anterior AAo wall appeared to have lost its normal structure and formed a dark-white mass (arrowheads). The red areas in the LA indicate blood flow from the RUPV. AAo: Ascending aorta; LA: Left atrium; RLPV: Right lower pulmonary vein; RUPV: Right upper pulmonary vein; SVC: Superior vena cava.

TEE revealed LA thrombi, which might have extended from the right lower pulmonary vein (RLPV) thrombi and white thrombi around the ostia of the RUPV, one of which seemed to be connected to linear white thrombi situated on the side of the anterior wall of the superior vena cava (SVC) and linear white thrombi penetrating the AAo wall, which existed as a mass on the AAo wall with AAo thrombi (Figure 1). There were blue areas on the AAo wall, which were situated between the end of the AAo wall and linear white thrombi, indicating disruption of the AAo wall. Scales were included in every imaging, which were located in the under parts of the enhanced CT images and on the upper and left sides of the TEE images. The distance between dots was 1 cm in both enhanced CT and TEE images.

Video images revealed rather vague, large, whitish thrombi (3 × 1.5 cm) around the central white areas in the AAo thrombi, and that the AAo thrombi moved with the heartbeat; however, the RUPV thrombi and linear thrombi did not move with the heartbeat (Video 1).

**Video 1:** TEE video images showing the cleft in the AAo wall and AAo thrombi The TEE revealed large thrombi (3 × 1.5 cm) in the AAo with white areas (1.2 × 0.4 cm) and RUPV thrombi, and the linear white thrombi seemed to connect to the RUPV thrombi and the white thrombi in the AAo thrombi. Overall, the linear white thrombi appeared to penetrate the AAo wall. The wall of the AAo, which seemed to be penetrated by linear white thrombi, exhibited a mass with no clear margin. The linear white thrombi appeared to penetrate the AAo wall, and blue blood flow appeared around the linear white thrombi in the mass, as highlighted in Figure 1 with arrowheads. Approximately 3 cm of the right anterior AAo lacked a normal structure. TEE: Transesophageal echocardiography; AAo: Ascending aorta; RUPV: Right upper pulmonary vein.

**Figure 2:**
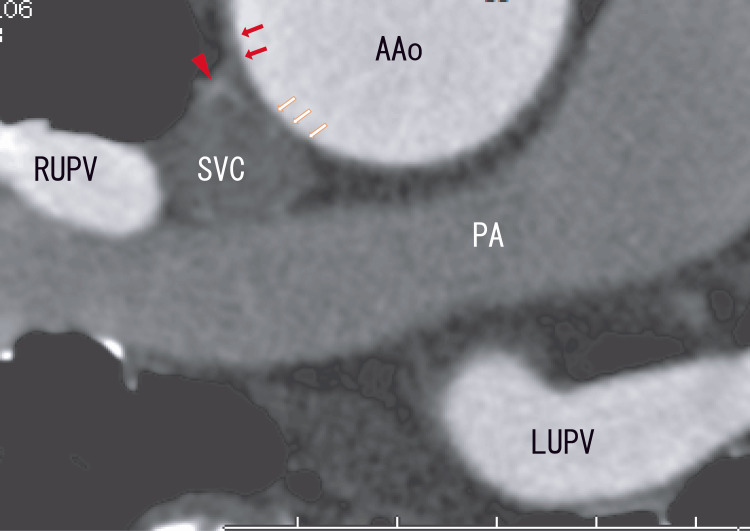
Axial enhanced computed tomography (CT) images showing the AAo and LA Axial enhanced CT images clearly revealed no cleft, no linear white thrombi, and no RUPV thrombi. There were some changes in the walls between the SVC and AAo (red arrows and white arrows). Small normal areas are visible between the white arrows and red arrows. There were white areas in the anterior parts of the SVC (arrowhead), indicating an arterial blood supply. AAo: Ascending aorta; LA: Left atrium; PA: Pulmonary artery; RUPV: Right upper pulmonary vein; SVC: Superior vena cava.

Cardiac CT did not reveal AAo thrombi (Figure 2), whose characteristics were similar to those of the LA thrombi that extended from the PV thrombi.

**Figure 3:**
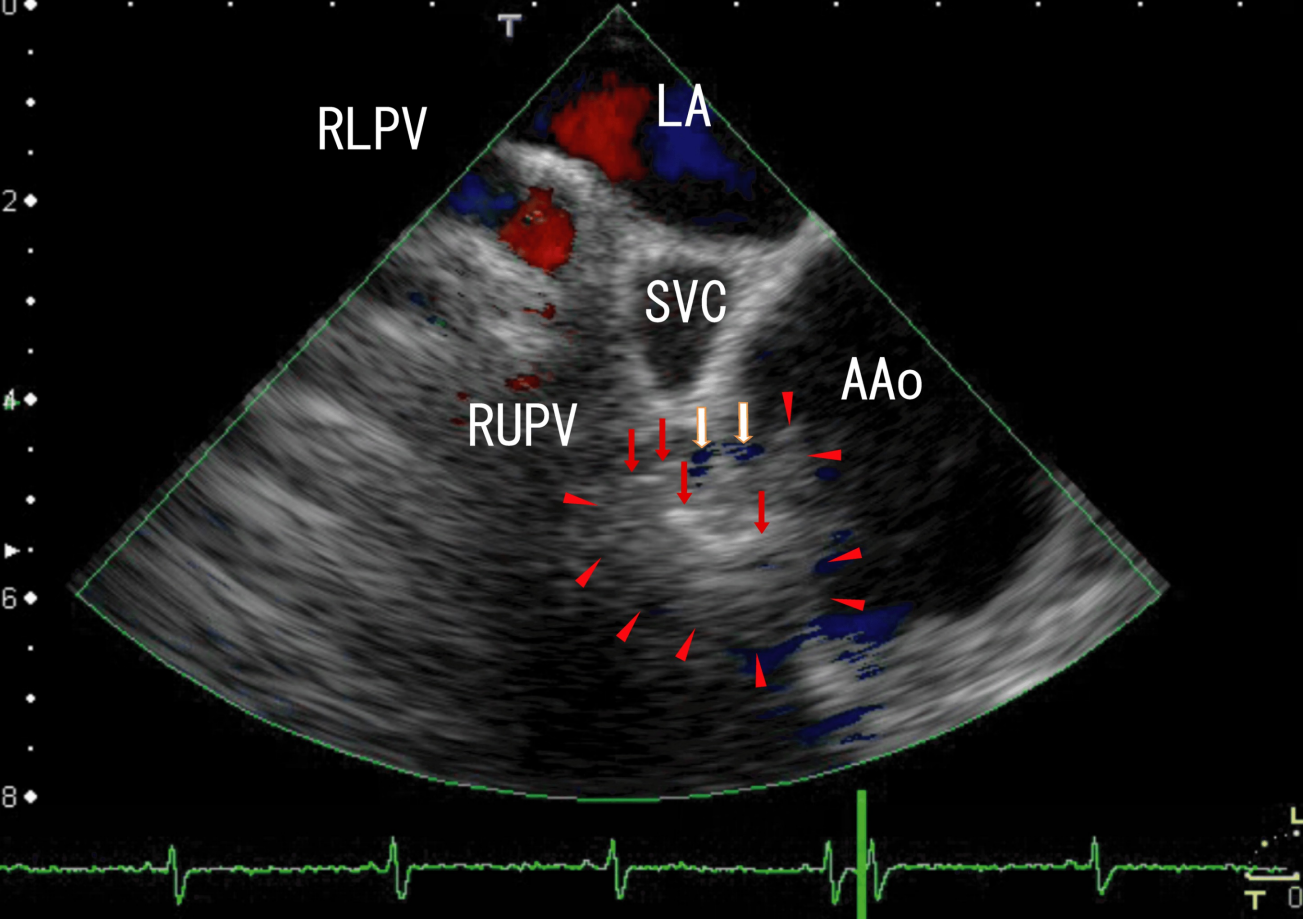
TEE images showing AAo thrombi and linear dark-white thrombi The TEE revealed thrombi in the AAo and linear dark-white thrombi extending from the RUPV into the AAo (red arrows), which had lost some clear white areas. Shorter linear white thrombi were observed in a similar position in Figure 1, which were situated in the mass on the AAo wall (arrowheads). Small blue areas were present between the AAo wall and the linear white thrombi on the AAo wall (white arrows), indicating a cleft in the AAo wall and penetration of the AAo wall by the thrombi. Approximately 2 cm of the right anterior AAo wall appeared to have lost its normal structure. A large mass (1.5 × 1.5 cm) was present on the AAo wall. The red areas in the LA indicate blood flow from the RUPV. TEE: Transesophageal echocardiography; AAo: Ascending aorta; LA: Left atrium; RLPV: Right lower pulmonary vein; RUPV: Right upper pulmonary vein; SVC: Superior vena cava.

The patient was treated with a standard dose of dabigatran (150 mg twice daily) for the first six months. The AAo thrombi partially resolved, and the linear white thrombi resolved and became vague (six months after the start of treatment), as shown in Figure 3 and Video 2. The distance of the discontinuous parts of the AAo wall became narrower. The serum D-dimer concentration was 0.5 μg/ml (normal: <1.0 μg/ml).

**Video 2:** TEE video images showing AAo thrombi and several dark-white areas The TEE video revealed large thrombi (1.5 × 1.5 cm) in the AAo with white areas (1.2 × 0.4 cm), and the linear dark-white areas seemed to connect to the AAo thrombi. A large cleft appeared in the AAo wall around the anterior SVC. In linear white thrombi, blood flow was sometimes detected as linear blue areas between upper and lower linear white thrombi. The wall of the AAo exhibited a mass with no clear margin, which seemed to be connected by linear white areas. Approximately 2 cm of the right anterior AAo wall lacked a normal structure. Additionally, the right posterior side of the AAo wall seemed to have vague and homogenous thrombi (1.5 × 1 cm). The LA thrombi did not move with the heartbeat but extended from the RLPV thrombi. The red areas in the LA indicate blood flow from the RUPV and the RLPV. TEE: Transesophageal echocardiography; AAo: Ascending aorta; LA: Left atrium; RLPV: Right lower pulmonary vein; RUPV: Right upper pulmonary vein; SVC: Superior vena cava.

**Figure 4:**
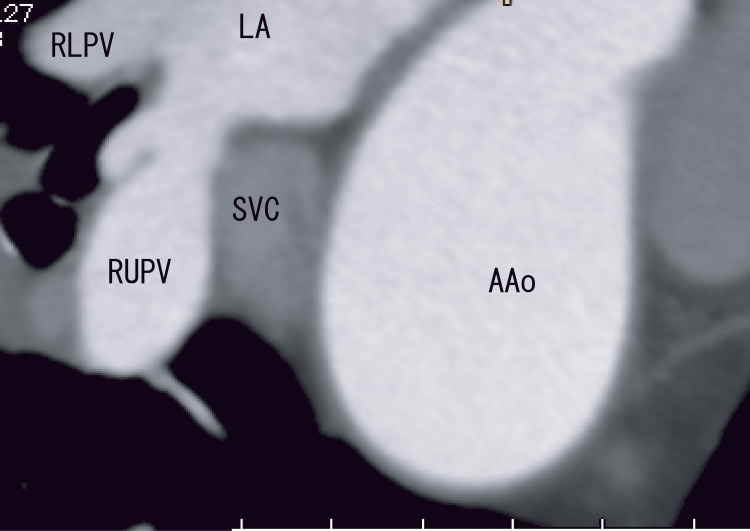
Oblique CT images showing the AAo and SVC Oblique enhanced CT images clearly revealed no cleft, no linear white thrombi, and no RUPV thrombi. There were a few changes in the walls between the SVC and AAo. The SVC has a vague boundary. The angle of these images is similar to that of the TEE images. AAo: Ascending aorta; LA: Left atrium; RLPV: Right lower pulmonary vein; RUPV: Right upper pulmonary vein; SVC: Superior vena cava.

Cardiac CT did not reveal AAo thrombi (Figure 4).

**Figure 5:**
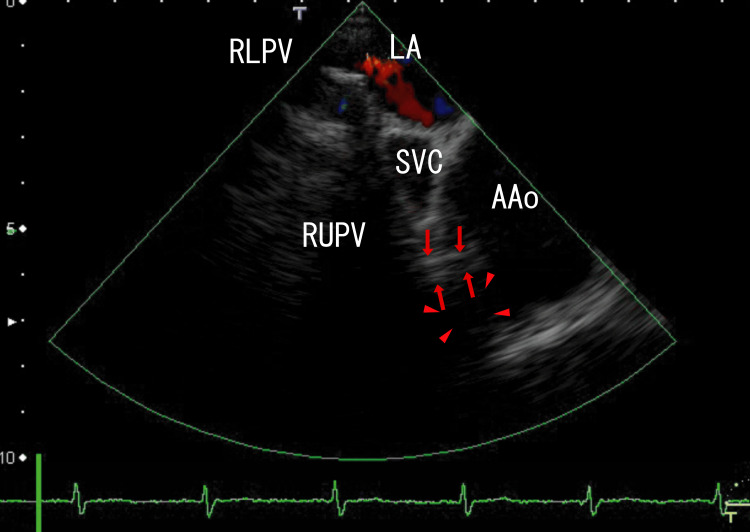
TEE images showing AAo thrombi and shortened linear dark-white thrombi The TEE revealed decreased thrombi in the AAo, but the linear dark-white thrombi from RUPV into the AAo (red arrows) might have disappeared. The large mass on the AAo wall disappeared. The red areas in the LA indicate blood flow from the RUPV; additionally, the blood flow in the RUPV appears as red areas, indicating that the RUPV thrombi were partially resolved. AAo: Ascending aorta; LA: Left atrium; RLPV: Right lower pulmonary vein; RUPV: Right upper pulmonary vein; SVC: Superior vena cava.

For the next eight months, we treated the patient with a decreased dosage of dabigatran (75 mg once a day) and a one-fourth dose of edoxaban (15 mg once a day) to avoid side effects. The TEE images revealed that the AAo thrombi were partially more resolved (1 × 0.5 cm) (Figure 5 and Video 3) 22 months from the start of treatment, and that the cleft in the AAo wall and linear white thrombi had disappeared. Only RUPV white thrombi could be identified as residual linear white thrombi. The blood flow from the RUPV became increasingly broader, indicating additional improvement in the RUPV thrombi. The serum D-dimer concentration was 0.7 μg/ml (normal: <1.0 μg/ml).

**Video 3:** TEE video images showing disappearance of the cleft and decreased AAo thrombi The TEE video revealed that the thrombi in the AAo were smaller (1 × 0.5 cm), and the linear white thrombi seemed to be dark and small. The white parts of the mass moved with the heartbeat. The red areas in the RUPV were enlarged, indicating resolution of the RUPV thrombi. The anterior parts of the AAo wall could not be clearly identified. The site of the mass had become a dark area, indicating the disappearance of the mass because of normalization of the blood supply. TEE: Transesophageal echocardiography; AAo: Ascending aorta; RUPV: Right upper pulmonary vein.

**Figure 6:**
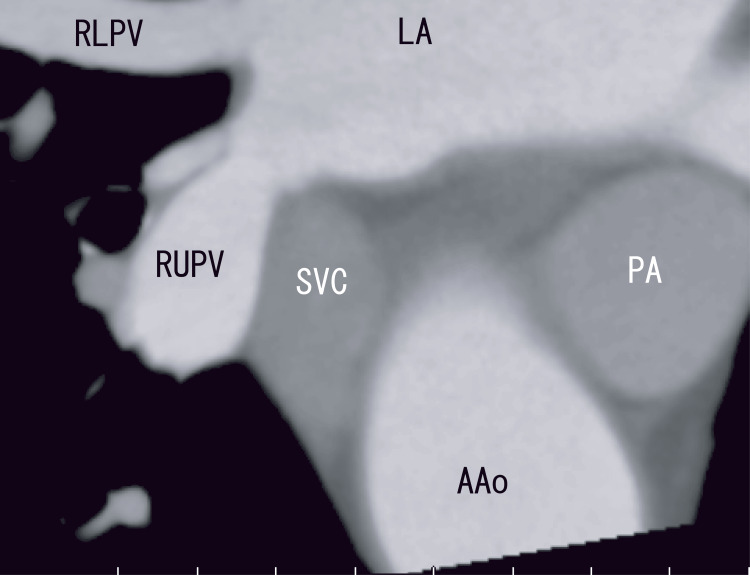
Oblique enhanced CT images showing the AAo and LA Oblique enhanced CT images clearly revealed no linear white thrombi and no changes in the AAo wall. The angle of these images is similar to that of the TEE images. AAo: Ascending aorta; LA: Left atrium; PA: Pulmonary artery; RLPV: Right lower pulmonary vein; RUPV: Right upper pulmonary vein; SVC: Superior vena cava.

Cardiac CT did not reveal AAo thrombi (Figure 6).

The main points of treatment are summarized in Table 1, and the initial laboratory results are summarized in Table 2.

**Table 1:** Main points of treatment DOACs: Direct oral anticoagulants; AAo: Ascending aorta; BNP: Brain natriuretic peptide.

Item	Start	6M	22M	Normal range
DOACs	-	Dabigatran	Early 8M: dabigatran; late 8M: edoxiban	
AAo thrombi (cm)	1.5 × 1.5	1.5 × 1.5	1 × 0.5	
The mass (cm)	1.5 × 1	1.5 × 1	1 × 0.3	
Line-like white thrombi (cm)	2 × 0.6 white	2 × 0.3 dark white	Disappeared	
The cleft (cm)	2 × (0.6~0.3)	2 × (0.3~0.2)	Disappeared	
Creatinine	0.56	0.55	0.68	0.47~0.79 mg/dL
BNP	22.4	31.9	26.3	<18.4 pg/mL
D-dimer	0.7	0.5	0.7	<1.0 μg/mL

**Table 2:** Initial laboratory results Hb: Hemoglobin; Plt: Platelet; BUN: Blood urea nitrogen; TG: Triglyceride; HDL-C: High-density-lipoprotein cholesterol; LDL-C: Low-density-lipoprotein cholesterol; CRP: C-reactive protein; FT4: Free thyroxine (3,5,3′,5′-tetraiodo-L-thyronine); FT3: Free 3,3′,5′-triiodo-L-thyronine; TSH: Thyroxine-stimulating hormone; ApoA1: Apolipoprotein A1; ApoA2: Apolipoprotein A2; ApoB: Apolipoprotein B; ApoC2: Apolipoprotein C2; ApoC3: Apolipoprotein C3; ApoE: Apolipoprotein E.

Item	Normal range	Results
Hb	14~18 g/dL	11.4
Plt	15~40 × 10^4^/μL	33.1
Fibrinogen	150~400 mg/dL	284
BUN	8~20 mg/dL	15.4
TG	35~150 mg/dL	72
HDL-C	40~100 mg/dL	66
LDL-C	70~140 mg/dL	67
CRP	<0.30 mg/dL	0.04
FT4	0.93~1.75 ng/dL	1.09
FT3	2.5~3.5 pg/mL	2.86
TSH	0.65~5.55 μU/mL	0.57
ApoA1	126~165 mg/dL	169
ApoA2	24.6~33.3 mg/dL	33.8
ApoB	66~101 mg/dL	60
ApoC2	1.5~3.8 mg/dL	5.5
ApoC3	5.4~9.0 mg/dL	12
ApoE	2.8~4.6 mg/dL	3.8

## Discussion

In our previous paper, we reported that RUPV thrombi and AAo thrombi were connected with linear white thrombi located beneath (anterior to) the SVC. These linear white thrombi approached the AAo wall and may affect abnormal changes in the AAo wall. The AAo wall in this area lost its normal structure and appeared as a mass of medial degenerative cells. The mass appeared homogeneous and had no clear structures. In particular, in the present case, there was a cleft in the abnormal mass, which could be the origin of AA/AD. The linear white thrombi appeared to penetrate the AA wall, organizing the anterior side of the cleft. These RUPV thrombi, the mass, and the linear white thrombi were not clearly identified by TEE or enhanced CT. The cleft was cured by treatment with dabigatran and edoxaban, with minimal side effects, which is described here for the first time. After AAo thrombi resolved, blood flow recovered around the abnormal AAo wall, and the cleft may have been cured. DOACs may recover arterial blood flow by resolving AAo thrombi.

Including the present case, I have reported three cases of similar AAo wall changes with similar mechanisms, indicating that these three component changes - RUPV thrombi, a mass on the AAo wall with AAo thrombi, and connecting linear white thrombi that are partially resolved using dabigatran and edoxaban with no adverse bleeding events - are not rare. The formation of these three structures could be related to the expression of certain genes; therefore, clarifying the function of these structures is important. Although previously atherosclerosis was considered to tend to increase distally from the ascending aorta, 60% of thoracic AA is observed in either the aortic root or the ascending aorta [9].

Medially degenerative cells in the mass may replace damaged heart cells to maintain normal heart function. If these supplier systems are inhibited, the heart cannot recover normally, and the heart might develop some diseases, such as dilated cardiomyopathy (DCM). Recently, cell sheet therapy using myoblasts [10] or induced pluripotent stem (iPS) cell-derived cardiomyocytes [11] was performed in patients with heart failure due to DCM or ischemic cardiomyopathy (ICM). iPS-derived cardiomyocytes might play a role in medial degeneration through unknown mechanisms. This is the first description of these hypotheses, and further studies are needed to clarify these relationships.

Research on retrieved thrombi has revealed two types of thrombus calcification: large and powder-like calcifications and large calcified thrombi containing many CD45+/CD68+/α-SMA+ cells [12], which can turn into myofibroblasts [13,14]. Myofibroblasts can transform into smooth muscle cells [15]. Myofibroblasts might transform into medially degenerative cells in the AAo wall mass​​​​. In the present case, RUPV thrombi and linear white thrombi did not exhibit accompanying shadows, indicating that no calcifications or myofibroblasts were present. The lack of myofibroblasts could be the cause of the cleft in the mass.

RUPV thrombi, AAo thrombi, and connecting linear white thrombi might result in the formation of a mass of medially degenerative cells. The mass might lack blood flow because of the presence of AAo thrombi, blocking blood flow to the mass. Linear white thrombi might contain vessel-like structures; however, such structures were not identified using enhanced CT, indicating abnormal arterial blood flow. The mass and linear white thrombi may lack appropriate blood flow, resulting in hypoxia, hypoglycemia, and carbon dioxide accumulation. Hypoglycemia affects mitochondrial function, and mitochondrial dysfunction is associated with hereditary thoracic aortic aneurysm development [16]. Mitochondria [17] and hypoxia [18,19] are associated with the degeneration process.

Edoxaban has been reported to decrease cerebral bleeding [20]. The underlying mechanism might be related to the improvement of cerebral artery damage, such as clefts. To clarify these mechanisms, more studies are needed.

Limitations

This case report describes a single case with​​​​​ some novel findings; therefore, the findings must be interpreted with caution. Changes in the AAo wall were observed only using TEE, and these changes could not be identified by cardiac CT. As a result, these changes should be examined with other modalities, including microscopy, and further genome studies are warranted​​​​​​.

## Conclusions

The abnormal AAo wall area was connected to RUPV thrombi through linear white thrombi, which appeared to penetrate the AAo wall and create a cleft. Treatment with dabigatran and edoxaban for 22 months partially resolved the AAo thrombi and linear white thrombi and repaired the cleft in the AAo. Dabigatran and edoxaban treatment have the possibility to prevent the occurrence of AA/AD; therefore, further studies are needed to confirm broader capabilities.
